# Retraction: Fibulin-4 is associated with prognosis of endometrial cancer patients and inhibits cancer cell invasion and metastasis via Wnt/β-catenin signaling pathway

**DOI:** 10.18632/oncotarget.28833

**Published:** 2026-04-17

**Authors:** Tiantian Wang, Mei Wang, Shuang Fang, Qiang Wang, Rui Fang, Jie Chen

**Affiliations:** ^1^Department of Maternal and Child Health, School of Public Health, Shandong University, Jinan 250012, China; ^2^Pharmacy Department, Shandong Provincial Hospital affiliated to Shandong University, Jinan 250012, China; ^3^Biochemistry and Molecular Biology, Georgetown University, Georgetown, Washington D.C 20057, USA; ^4^Department of Obstetrics and Gynecology, The Second Hospital affiliated to Jilin University, Jilin 130000, China; ^5^Clinical Medicine, School of Medicine, Shandong University, Jinan 250012, China

**This article has been retracted:** Oncotarget has completed its investigation of this paper. It was determined that the paper contains multiple instances of image duplication and overlap, both internal to the paper and external with other published works. Specifically, we identified the following issues:

Internal Duplications and Overlaps:

Figure 2D transwell assay: HEC-1A cells image overlaps with Figure 7A (ISK-1 cells) non-infected and NC images.
Figure 2E transwell assay: HEC-1A cells image is a duplicate of Figure 8A NC (ISK-1 cells).
Figure 7A transwell assay: noninfected and NC images show overlap.
Figure 10A western blot: N-cadherin expression in KLE-28 cells image is a duplicate of Figure 13A image of N-cadherin in ISK-1 cells.
Figure 13A western blot: vimentin expression in ISK-23 cells image is a duplicate of Figure 13A b-catenin expression in KLE-28 cells.
Figure 5B immunocytochemistry staining: two images (noninfected and NC) overlap in the ISK-1 panel and the ISK-1/NC image is a duplicate of Figure 4A image of ISK-1/Fibulin-4.
Figure 12 (ISK-23 b-catenin and CyclinD1) are duplicates of Figure 7A (Ishikava vimentin and N-cadherin), respectively.
Supplemental Figure 2D (KLE-28) is a duplicate of Figure 8A (ISK-23 NC).
Supplemental Figure 2C (KLE-28) is a duplicate of Figure 8A (Non-infected ISK-1).

External Duplications and Manipulations:

Figure 2C colony formation assay: HEC-1A/NC image duplicates NC for HOS-1 cells in Figure 6C of reference [1], where images overlap after rotation.
Figure 10A and 11A western blots: KLE-28/N-cadherin and ISK-1/E-cadherin are manipulated duplicates of the blot for HOS-1 protein Slag in Figure 9A of reference [1].
Figure 3A endometrial cells immunocytochemistry with Fibulin 4 is a duplicate of Figure 4A in reference [2] where endometrial cells are stained for HMGB1.
Figure 11A western blot: KLE-1/N-cadherin image is a duplicate of Figure 7C E-cadherin in reference [2].
Figure 13A western blot: ISK-23/vimentin image is a duplicate of b-catenin image of KLE-28 cells, KLA-28 N-cadherin blot is a duplicate of CDK2 in reference [3].
Figure 13B western blot: GAPDH blot of ISK1 cells is a duplicate of b-actin blot of 786-o cells in Figure 7B in reference [3] and KLE-1/vimentin blot is a duplicate of 786-o/Bax image from [3].
Supplementary Figure 2E “Photograph of xenografts formed in nude mice after subcutaneous inoculation” was reused in a 2018 article [4] of the corresponding author Jie Chen.
While the authors did provide some original images, the number and nature of these issues remain a serious concern regarding the integrity and reliability of the reported data. Given the extent of the detected image duplications, the editorial decision has been made to retract this publication. The authors were contacted regarding this decision but did not agree to the retraction.

Original article: Oncotarget. 2017; 8:18991–19012. 18991-19012. https://doi.org/10.18632/oncotarget.15086

## References

[1] Wang S, Zhang D, Han S, Gao P, Liu C, Li J, Pan X. Fibulin-3 promotes osteosarcoma invasion and metastasis by inducing epithelial to mesenchymal transition and activating the Wnt/β-catenin signaling pathway. Sci Rep. 2017; 7:6215. 10.1038/s41598-017-06353-2. . Retraction in: Sci Rep. 2023; 13:11641. DOI: 10.1038/s41598-023-38918-9 PMID: 37468568 28740094 PMC5524709

[2] Luan X, Ma C, Wang P, Lou F. HMGB1 is negatively correlated with the development of endometrial carcinoma and prevents cancer cell invasion and metastasis by inhibiting the process of epithelial-to-mesenchymal transition. Onco Targets Ther. 2017; 10:1389–402. 10.2147/OTT.S123085. 28424555 PMC5344438

[3] Deng W, Han W, Fan T, Wang X, Cheng Z, Wan B, Chen J. Scutellarin inhibits human renal cancer cell proliferation and migration via upregulation of PTEN. Biomed Pharmacother. 2018; 107:1505–13. 10.1016/j.biopha.2018.08.127. 30257368

[4] Li J, Qi C, Liu X, Li C, Chen J, Shi M. Fibulin-3 knockdown inhibits cervical cancer cell growth and metastasis in vitro and in vivo. Sci Rep. 2018; 8:10594. 10.1038/s41598-018-28906-9. . Retraction in: Sci Rep. 2023; 13:11162. DOI: 10.1038/s41598-023-38389-y PMID: 37429930 30006571 PMC6045626

